# A Beneficial Effect of Low-Dose Aspirin in a Murine Model of Active Tuberculosis

**DOI:** 10.3389/fimmu.2018.00798

**Published:** 2018-04-23

**Authors:** Vera Marie Kroesen, Paula Rodríguez-Martínez, Eric García, Yaiza Rosales, Jorge Díaz, Montse Martín-Céspedes, Gustavo Tapia, Maria Rosa Sarrias, Pere-Joan Cardona, Cristina Vilaplana

**Affiliations:** ^1^Experimental Tuberculosis Unit (UTE), Fundació Institut Germans Trias i Pujol (IGTP), Universitat Autònoma de Barcelona (UAB), Badalona, Spain; ^2^Carl-von-Ossietzky University Oldenburg, Oldenburg, Germany; ^3^Pathology Department, Hospital Universitari Germans Trias i Pujol (HUGTIP), Universitat Autònoma de Barcelona (UAB), Badalona, Spain; ^4^Innate Immunity Group, Fundació Institut Germans Trias i Pujol (IGTP), Badalona, Spain; ^5^Centro de Investigación Biomédica en Red de Enfermedades Hepáticas y Digestivas (CIBEREhD), Madrid, Spain; ^6^Centro de Investigación Biomédica en Red de Enfermedades Respiratorias (CIBERES), Madrid, Spain

**Keywords:** aspirin, host-directed therapies, non-steroidal anti-inflammatory drugs, tuberculosis, *Mycobacterium tuberculosis*, mouse model

## Abstract

An excessive, non-productive host-immune response is detrimental in active, chronic tuberculosis (TB) disease as it typically leads to tissue damage. Given their anti-inflammatory effect, non-steroidal anti-inflammatory drugs can potentially attenuate excessive inflammation in active TB disease. As such, we investigated the prophylactic and therapeutic effect of low-dose aspirin (LDA) (3 mg/kg/day), either alone or in combination with common anti-TB treatment or BCG vaccination, on disease outcome in an experimental murine model of active TB. Survival rate, bacillary load (BL) in lungs, and lung pathology were measured. The possible mechanism of action of LDA on the host’s immune response was also evaluated by measuring levels of CD5L/AIM, selected cytokines/chemokines and other inflammatory markers in serum and lung tissue. LDA increased survival, had anti-inflammatory effects, reduced lung pathology, and decreased bacillary load in late-stage TB disease. Moreover, in combination with common anti-TB treatment, LDA enhanced survival and reduced lung pathology. Results from the immunological studies suggest the anti-inflammatory action of LDA at both a local and a systemic level. Our results showed a systemic decrease in neutrophilic recruitment, decreased levels of acute-phase reaction cytokines (IL-6, IL-1β, and TNF-α) at late stage and a delay in the decrease in T cell response (in terms of IFN-γ, IL-2, and IL-10 serum levels) that occurs during the course of *Mycobacterium tuberculosis* infection. An anti-inflammatory milieu was detected in the lung, with less neutrophil recruitment and lower levels of tissue factor. In conclusion, LDA may be beneficial as an adjunct to standard anti-TB treatment in the later stage of active TB by reducing excess, non-productive inflammation, while enhancing Th1-cell responses for elimination of the bacilli.

## Introduction

Tuberculosis (TB), which is a chronic infectious disease caused by *Mycobacterium tuberculosis*, ranks among the top 10 causes of death worldwide (1). In a clinical context, human TB presents with a wide spectrum of disease, ranging from asymptomatic infection to active TB disease (2). Patients can usually be divided into having latent TB infection or suffering from active TB disease, depending on whether they show clinical signs of the disease or not (2). Patients with co-morbidities that induce a pro-inflammatory milieu, such as diabetes mellitus and tobacco smoking, an impaired immune response, such as the elderly, children, people relying on immunosuppressive therapy, such as anti-TNFs (3) or with immunodeficiencies, such as those co-infected with HIV (1), are particularly at risk of developing active TB disease once infected with *M. tuberculosis*.

The current approach to treating TB entails antimicrobial drugs that target the mycobacteria (4). Thus, the standard 6-month treatment for TB patients consists of a four-drug regimen containing rifampicin, isoniazid, pyrazinamide, and ethambutol (4). Multi-drug resistant tuberculosis (MDR-TB), defined as resistance to at least two first-line drugs isoniazid and rifampicin, and extensively-drug resistant tuberculosis, where additional resistance to any fluoroquinolone or any of the injectable second-line aminoglycosides occurs, is an emerging challenge in the treatment of TB (1, 5). In 2015, the WHO reported an incidence of 450,000 new cases of MDR-TB worldwide, and it has been reported in virtually every country (1). One strategy that has been suggested to more effectively fight against TB, MDR-TB, and TB/HIV involves drugs that target host immune functions as adjuvants to classic antimicrobial treatment rather than focusing on the bacteria themselves (6). By not targeting the infecting microorganisms, these drugs have the potential advantage of not selecting TB drug resistance. The majority of these so-called host-directed therapies (HDTs) (7), which include non-steroidal anti-inflammatory drugs (NSAIDS) (8, 9), aim to balance host immune responses in order to decrease damage and are potentially able to achieve clinical improvement and decreased morbidity and mortality. Pathological immune reactions in the host, such as an insufficient or excessive inflammatory response, the latter of which leads to severe tissue damage, are considered to be a major cause of failure of current TB treatments (7).

Upon infection, *M. tuberculosis* is phagocytosed by alveolar macrophages, where it prevents phagosome–lysosome fusion and elimination by the lysosome (10). Infected antigen-presenting cells secrete various cytokines and chemokines to activate the innate immune response and influx of neutrophils (11, 12). Neutrophils usually represent a protective immune response during early infection by secreting oxidizing and hydrolytic agents that target the bacteria (11). However, although this neutrophil-dominated inflammation is beneficial in acute infections, where a fast and strong immune response can potentially lead to control or even clearance of the bacilli, it can be detrimental when non-productive and excessive, as described above, in the context of HIV co-infection (13), or simply in chronic infection (11). In active, chronic TB disease, an early and strong immune response has been reported to destroy delicate neighboring host tissues, thus leading to necrosis and resulting in cavitation, which facilitates spread of the bacilli, rather than containing *M. tuberculosis* replication and the continuous, excessively aggressive immune response (14). Attenuating the excessive host inflammatory response in active TB disease might thus be vital for treatment and disease outcome (11). Given their anti-inflammatory effect, NSAIDS can potentially attenuate neutrophil-mediated inflammation in TB (9, 8). Similarly, cyclooxygenase (COX) inhibitors have also been suggested to have potential therapeutic applications in other infections, such as in the control of parasite replication and dissemination in Chagas disease (15–17), in *Leishmania major* infection (18) and in pneumonia and pneumococcal-influenza co-infection (19).

In this study, we aimed to investigate the effect of low-dose aspirin (AAS) (LDA), administered alone or as an adjunct to common anti-TB treatment with the standard antibiotic treatment-regimen for human patients or with preventative BCG vaccination, in a murine model of active TB. Disease outcome was quantified in terms of survival rate, bacillary load (BL) in lungs, and lung pathology. LDA has previously been shown to prolong survival and enhance control of BL in the late stages of TB (12) and was, therefore, to confirm this finding. AAS is a salicylate that inhibits the two isoforms of COX in an irreversible manner, while leaving LOX activity unaffected (20, 21). The LOX pathway results in the synthesis of lipoxins (LX), which are known to be immunoresolvents with a potent anti-inflammatory effect and potential antimicrobial properties (22). The resulting synthesis of LX promotes the switching from a pro-inflammatory to anti-inflammatory milieu, with a decrease in pro-inflammatory cytokines and less neutrophil recruitment. Inhibition of the COX pathway also has an impact on vascularization and is widely used in the prevention of cardiovascular disease and stroke (21).

In light of the above, we decided to explore whether the described beneficial effects of LDA on survival rate, pathology, and BL in lungs in active TB are mediated by an anti-inflammatory effect, as previously found with ibuprofen (23). To assess the potential anti-inflammatory effect of LDA, we measured immunological profiles in serum and lungs in a murine model of active TB. Our specific objectives were: (1) to provide further evidence for the described beneficial effects of LDA on disease outcome in a murine model of active TB, when given prophylactically or therapeutically, by assessing survival rate, pulmonary BL, and lung pathology; (2) to investigate whether the potential beneficial effects are mediated by an anti-inflammatory mechanism of action; (3) to draw conclusions regarding the general immune responses in TB (as possible target for HDTs) by comparing immunological profiles in infected animals and non-infected negative control mice.

## Materials and Methods

### Experimental Design

A murine model of active TB based on a single intravenous (i.v.) infection [4 × 10^4^–2 × 10^5^/mL colony forming units (CFU)/mL per mouse] with *M. tuberculosis* H37Rv Pasteur strain in C3HeB/FeJ mice was used, as described previously (15). Animals (*n* = 164) were divided into five experiments (see Table 1):

(a) To evaluate the prophylactic effect of LDA: Experiments 1 and 2. AAS was given from 1 week before infection and disease outcome compared in aspirin-treated and non-treated animals.
(b) To evaluate the therapeutic effect of LDA: Experiments 3 to 5. In Experiment 3 (*n* = 24), disease outcome was compared in LDA-treated and non-treated animals. In Experiment 4, LDA was evaluated as a coadjuvant with BCG vaccination (*n* = 36). Mice were subcutaneously vaccinated with sham (SF) or BCG 10^6^ CFU/animal [ImmunoCyst^®^ BCG, Sanofi Pasteur (batch E-5-C4042A)] alone or in combination with therapeutical LDA (administered 2 weeks post-challenge). The results for the BCG- and BCG + AAS-treated groups were compared with those obtained for the sham group. The endpoint was established at week 18 after the start of the experiment as these BCG-vaccinated or BCG-vaccinated and AAS-treated mice did not die. Experiment 5 (*n* = 24) was designed to assess the therapeutic effect of LDA in combination with RIMSTAR, the standard antibiotic combination used in human TB treatment.

**Table 1:** Experimental design of the five experiments included in the study.

Experimental design							
Experiment 1: prophylaxis, survival experiment	Group	W-1	W1	W3→			
	
	Control	–	Day 0: infection	–			
	
	AAS	AAS 3 mg/kg/day	Day 0: infection. AAS 3mg/kg/day	AAS 3 mg/kg/day continued until death			

Experiment 2: prophylaxis, scheduled timepoints	Group	W-1	W1	W2	W3	W4	
	
	Control	–	Day 0: infection	Day 14: euthanasia of 6 mice; serum collection	Day 21: euthanasia of 6 mice; serum collection	Day 28: euthanasia of 6 mice; serum collection	
	
	AAS	AAS 3 mg/kg/day	Day 0: infection. AAS 3 mg/kg/day	Day 14: euthanasia of 6 mice; serum collection. AAS 3 mg/kg/day	Day 21: euthanasia of 6 mice; serum collection. AAS 3 mg/kg/day	Day 28: euthanasia of 6 mice; serum collection. AAS 3 mg/kg/day	

Experiment 3: therapeutics, survival experiment	Group	W-1	W1	W2–W4	W5	W6	W7→
	
	Control		Infection	–	–	–	–
	
	AAS		Infection	–	AAS 3 mg/kg/day	AAS 3 mg/kg/day	–

Experiment 4: therapeutics, synergy with BCG	Group	W-1	W1	W2–6	W7	W8	W9→
	
	Sham		Vacc.		Infection		
	
	BCG		Vacc.		Infection		
	
	BCG + AAS		Vacc.		Infection		AAS 3 mg/kg/day continued until death

Experiment 5: therapeutics, synergy with Rimstar	Group	W-1	W1	W2–4	W5	W6	W7→
	
	RIMSTAR		Infection		RIMSTAR	RIMSTAR	RIMSTAR continued until death
	
	RIMSTAR + AAS		Infection		RIMSTAR + AAS 3 mg/kg/day	RIMSTAR + AAS 3 mg/kg/day	RIMSTAR continued until death

*AAS, aspirin; BCG, bacille Calmette–Guerin vaccine; Vacc., vaccination; RIMSTAR, rifampicin 150 mg + isoniazid 75 mg + pyrazinamid 400 mg + ethambutol 275 mg, adjusted to mouse weight; W, week; W-1, one week before challenge; W2, 3, 4, 5, 6, etc., means weeks after challenge*.

### The Murine Model of Acute Active TB (C3HeB/FeJ Mice)

The C3HeB/FeJ murine model of acute active TB disease in humans was selected due to its suitability for investigating the effect of therapeutical strategies on disease outcome and immune mechanisms during active TB disease with excess inflammation and granuloma formation (12).

### Endpoints to Assess the Impact of LDA on Disease Outcome

The effect of LDA on disease outcome was measured in terms of survival rate (all experiments except number 2; *n* = 108), BL in lungs at different timepoints (days 14, 21, and 28 post-infection; *n* = 48) and lung pathology analysis (histometry; *n* = 138) (Table 2). For the BL assessment, samples of lung lobes from animals from experiment 2 were collected, homogenized, and several dilutions plated on nutrient Middlebrook 7H11 agar (BD Diagnostics, Spark, USA). The number of CFU was counted after incubation for 28 days at 37°C and the results expressed as CFU/mL.

**Table 2:** Overview of the experiments and methods employed.

Experiment	Experimental group	Treatment	No. of mice	Survival assessed	BL assessed	No. of slides for histometry	Serum samples for Luminex and ELISA
1	Positive control	–	12	Yes	No	12	No
	AAS	LDA	12			11	No

2	Positive control	–	24	No	Yes	18	Yes
	AAS	LDA	24			13	Yes

3	Positive control	–	12	Yes	No	12	No
	AAS	LDA	12			12	No

4	sham	sham	12			12	No
	BCG	BCG	12			12	No
	BCG + AAS	BCG + LDA	12	12	No

5	Rimstar	Rimstar	12	Yes	No	12	No
	Rimstar + AAS	Rimstar + LDA	12			12	No

Negative ct	Negative ct	–	6	No	No	No	Yes, only Luminex

*AAS, aspirin; LDA, low-dose aspirin 3 mg/kg/day; BCG, bacille Calmette–Guerin vaccine; RIMSTAR, rifampicin 150 mg + isoniazid 75 mg + pyrazinamid 400 mg + ethambutol 275 mg, adjusted to mouse weight; Negative ct, negative control, healthy animals*.

For the assessment of lung pathology, histological lung sections from C3HeB/FeJ mice from all experimental groups were analyzed by histometry. Lungs were fixed in 10% buffered formalin, embedded in paraffin and 5-µm sections stained with hematoxylin-eosin (HE) stain for histometric analysis. All slides were scanned at a resolution of 300 dpi (dots per inch), and scanned images analyzed using NIS-Elements Documentation 3.0 Software (by Nikon Instruments; Shinjuku, Tokio, Japan). The percentage of affected area in relation to total lung area was measured.

### Methods to Assess the Impact of LDA on Immunological Profiles in Serum and Lung Tissue

The immunological profiles in sera from the experimental groups were assessed in Experiment 2 (Table 2). Ten selected cytokines and chemokines (G-CSF, KC, MIP-2, IL-1α, IL-1β, IL-6, TNFα, IL-2, IFNγ, and IL-17) were measured in serum samples obtained at days 14 (week 2), 21 (week 3), and 28 (week 4) post-infection, using the Luminex MILLIPLEX^®^ MAP Kit, Mouse Cytokine/Chemokine Magnetic Bead Panel, 96-Well Plate Assay (EMD Millipore). Six non-infected mice were used as negative control in the Luminex assay. Circulating CD5L/AIM protein was assessed using the CircuLex Mouse AIM/CD5L/Spα ELISA Kit (MBL International Corporation, Woburn, USA) and the same sera samples. Both kits were used in accordance with their manufacturer’s instructions.

Immunohistochemical stains on paraffined sections were performed using the ultraView DAB (Ventana Medical Systems) in accordance with the manufacturer’s protocol. Monoclonal antibodies against TNF-α (rabbit polyclonal ab6671; Abcam, dilution 1:100), STAT1 (phospho S727) (rabbit monoclonal EPR3146; Abcam, dilution 1:500), myeloperoxidase (MPO) (rabbit polyclonal ab45977, Abcam, dilution 1:500), arginase 1 (rabbit polyclonal ab91279, Abcam, dilution 1:200), iNOS (rabbit polyclonal ab15323, Abcam, dilution 1:100), CD5L (dilution 1:50), tissue factor (TF) (rabbit monoclonal ab151748, Abcam, dilution 1:200) were used. Granulomas were imaged with a camera and NIS-Elements Documentation 3.0 Software connected to a microscope (Nikon Instruments; Shinjuku, Tokyo, Japan). As individual tissue sections from different animals with active TB contain multiple granulomas, we decided to image regions from five different granulomas with representative features, and to use them to analyze the IHC staining (5 per experimental group and timepoint). Images were acquired as TIFF-format images and analyzed with ImageJ (available at https://imagej.nih.gov/ij/). The median stained area for each antibody was graphically represented as square pixels.

### Graphics and Statistics

Data are shown as median ± SEM. A non-parametric comparison (Mann–Whitney test) was used for comparisons between LDA and control groups (GraphPad Software v6.0). Statistically significant differences are designated as follows: *p < 0.05; **p < 0.01; ***p < 0.001.

## Results

### Survival Rate

**Figure 1:**
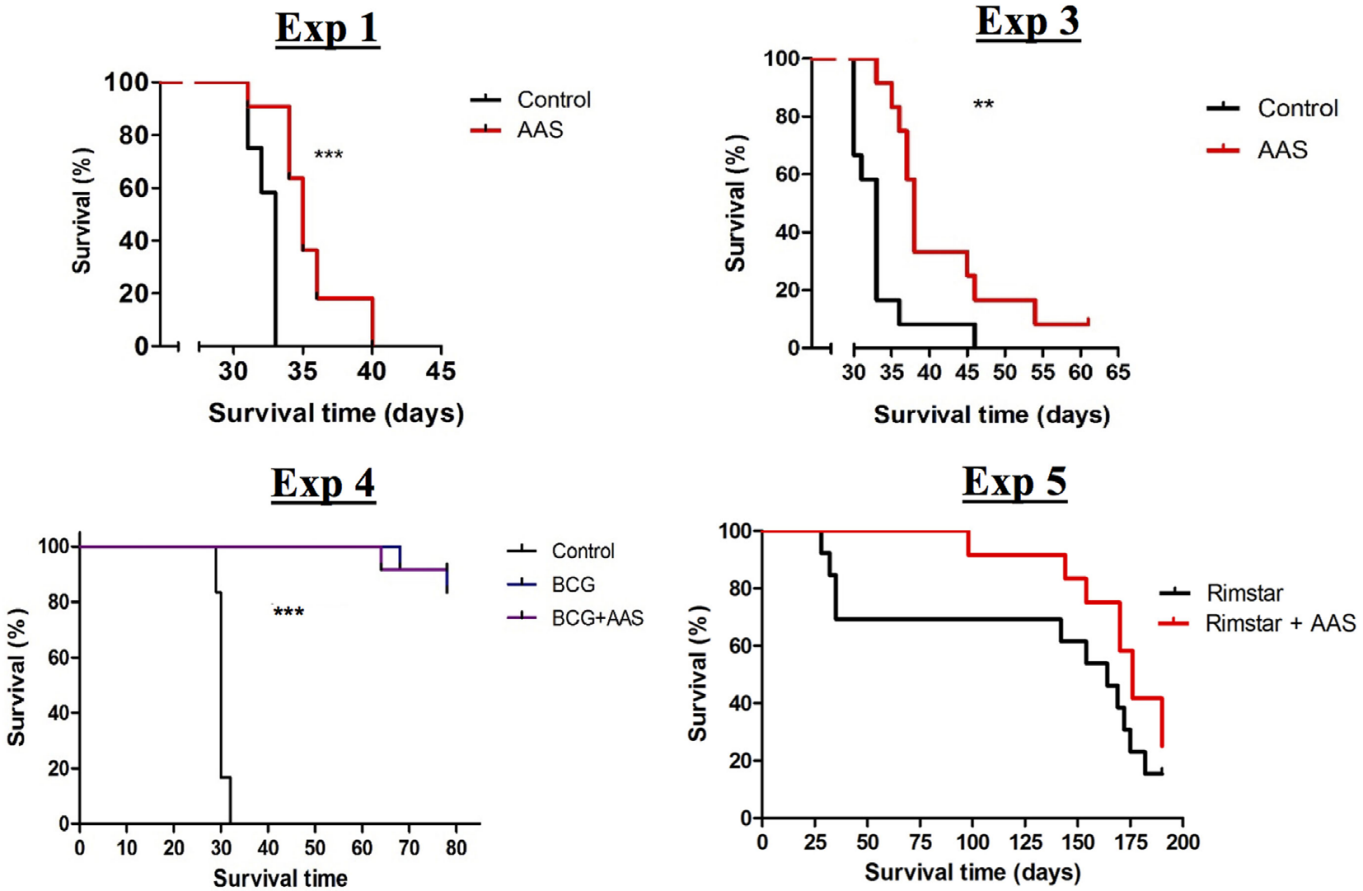
Survival assessment. Curves for the experimental groups achieved in the four experiments in which the impact of treatment on survival was assessed. Abbreviations: AAS, aspirin; BCG, bacille Calmette–Guerin vaccine; Vacc., vaccination; RIMSTAR, rifampicin 150 mg + isoniazid 75 mg + pyrazinamid 400 mg + ethambutol 275 mg, adjusted to mouse weight.

Low-dose aspirin increased the survival rate when compared to controls in all experiments in which survival was assessed (experiments 1, 3, 4, and 5) (Figure 1). Both prophylactic and therapeutic treatment with LDA (experiments 1 and 3) increased survival compared to non-treated controls in a statistically significant manner (*p* < 0.001 and *p* = 0.0032, respectively). Both BCG vaccination alone and in combination with LDA (experiment 4) showed a statistically significant increase in survival compared to the control group (*p* < 0.001), although no additional effect of LDA was found, at least until termination of the experiment. Two weeks of LDA in addition to antibiotic treatment (experiment 5) increased survival compared to RIMSTAR-only treated animals, although the differences between the survival curves were not statistically significant, at least not with the number of animals included in each treatment group (*n* = 12).

### BL in Lungs

**Figure 2:**
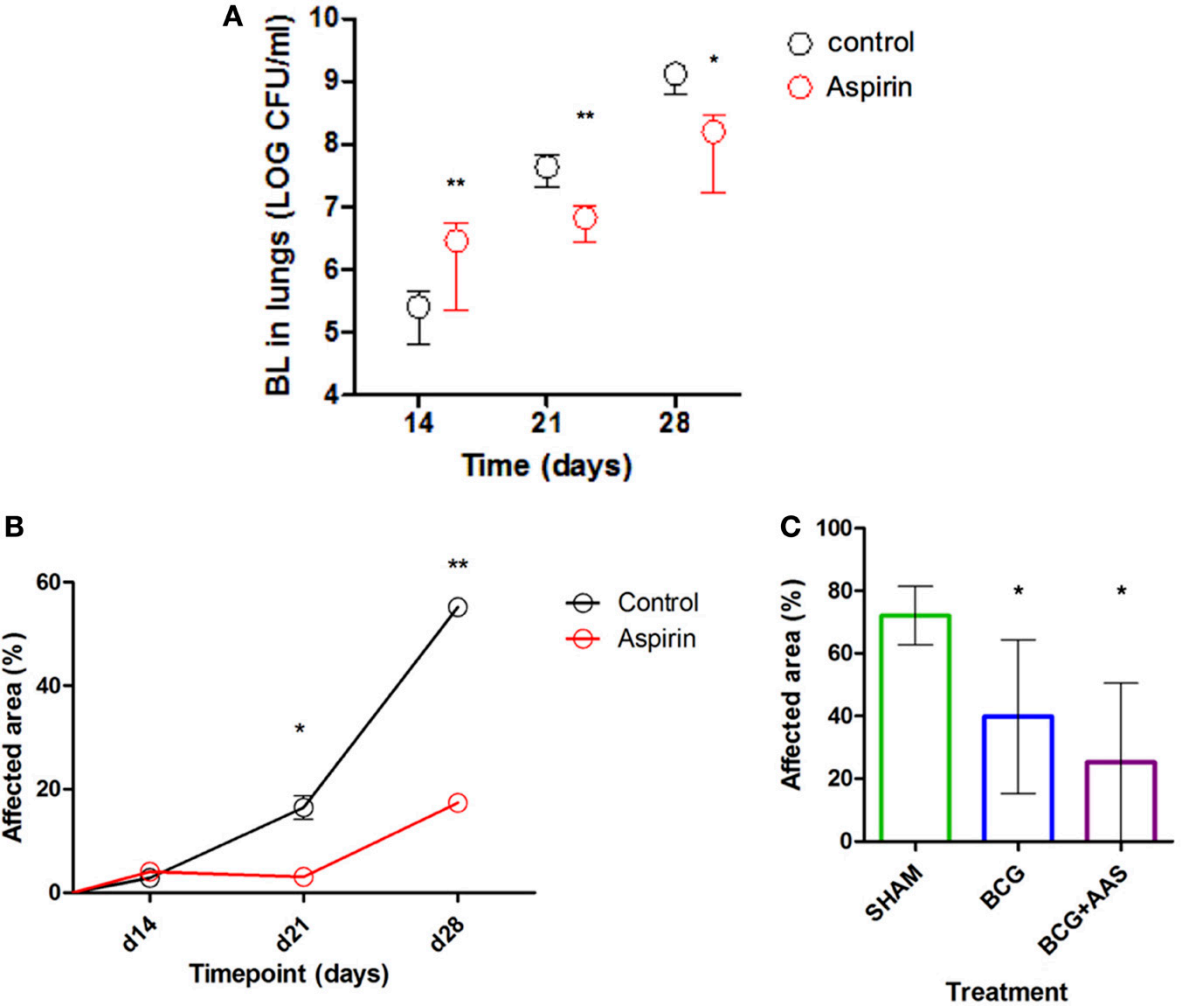
Impact of AAS on the BL and histometry. AAS, aspirin; BCG, bacille Calmette–Guerin vaccine; BL, bacillary load. Panel **(A)** shows the impact of AAS on the BL with time post-challenge. Panels **(B,C)** show the impact of AAS on the histometry in terms of affected lung area, both alone [**(B)**: experiment 2] and in combination with BCG vaccine [**(C)**: experiment 4].

Prophylactic treatment with LDA (experiment 2) showed a statistically significant increase in BL on day 14 post-infection (*p* = 0.0022), but a statistically significant decrease at later stages (day 21, *p* = 0.0050; day 28, *p* = 0.0152) when compared to controls (Figure 2A).

### Lung Pathology (Histometry)

Histological lung sections obtained from mice from all experiments were stained with HE-stain and analyzed in terms of granulomatous area in lungs (%). A statistically significant increase in lung pathology in both treatment groups over the course of infection was seen in those animals used to evaluate the BL at different timepoints (experiment 2). This increase was statistically significantly delayed and mitigated by preventive treatment with LDA only. As shown in Figure 2B, the % affected area was significantly reduced in LDA-treated mice compared to controls in late-stage infection (day 21, *p* = 0.0286; day 28, *p* = 0.0286).

BCG vaccination was found to prevent excess lung pathology compared to sham (*p* = 0.0169) (Figure 2C), keeping lung pathology very low at least until the end of the experiment at 4 months post-infection [mean of 39.78%; SD 24.5% (BCG group) and 72.07%; SD 9.335% (sham group)]. Adjunctive LDA treatment had an additional beneficial impact on this effect, although this was not statistically significant when compared to BCG alone.

### Immunological Studies

**Figure 3:**
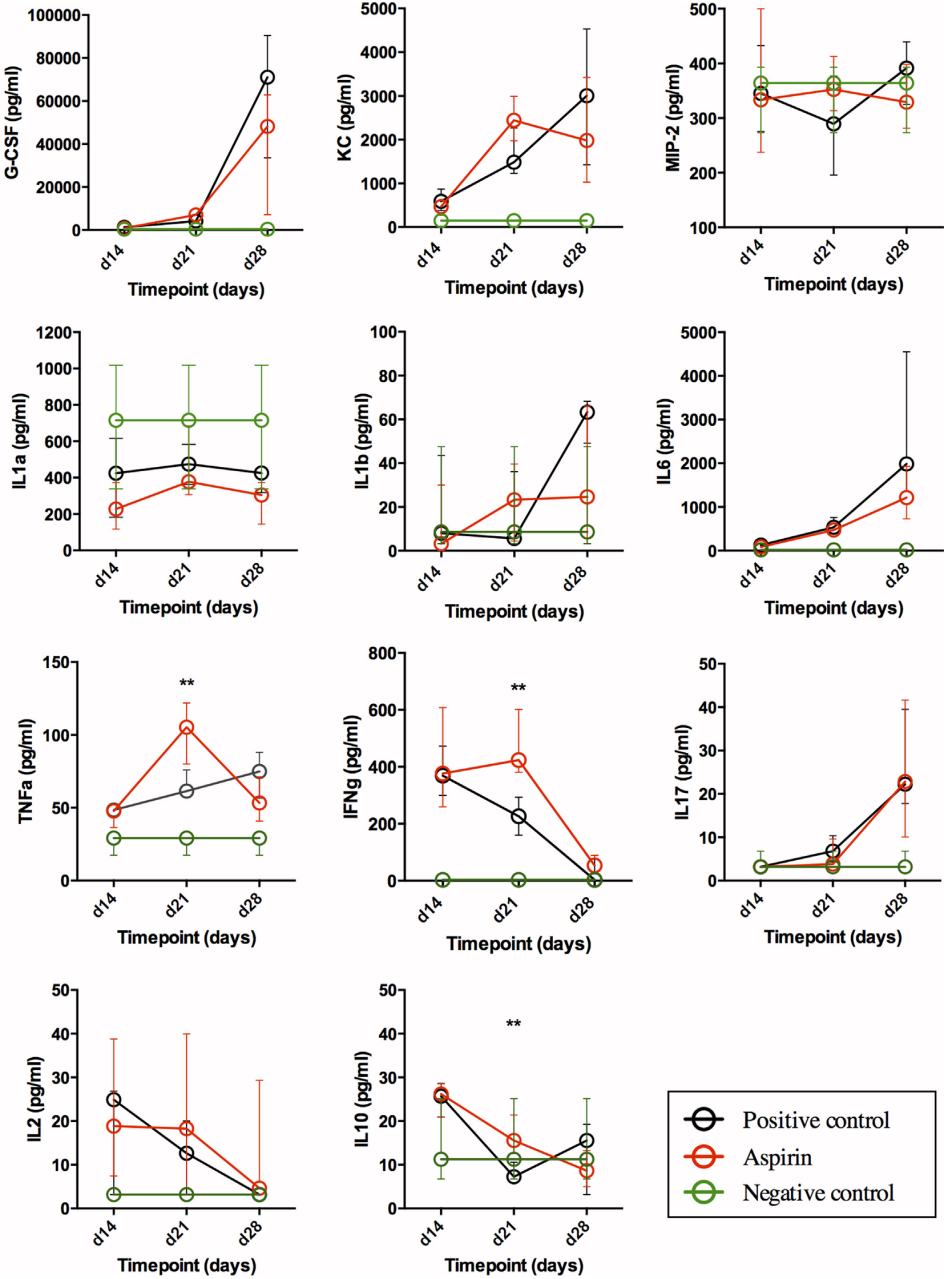
Impact of AAS on serum cytokine/chemokine levels. All data are presented in pg/mL. Abbreviations: AAS, aspirin; G-CSF, granulocyte-colony stimulating factor; KC, chemokine (C-X-C motif) ligand 1 (CXCL-1); MIP-2, macrophage inflammatory protein 2-alpha [also named chemokine (C-X-C motif) ligand 2 (CXCL2)]; IL, interleukin; TNFa, tumor necrosis α; IFNg, interferon γ.

**Figure 4:**
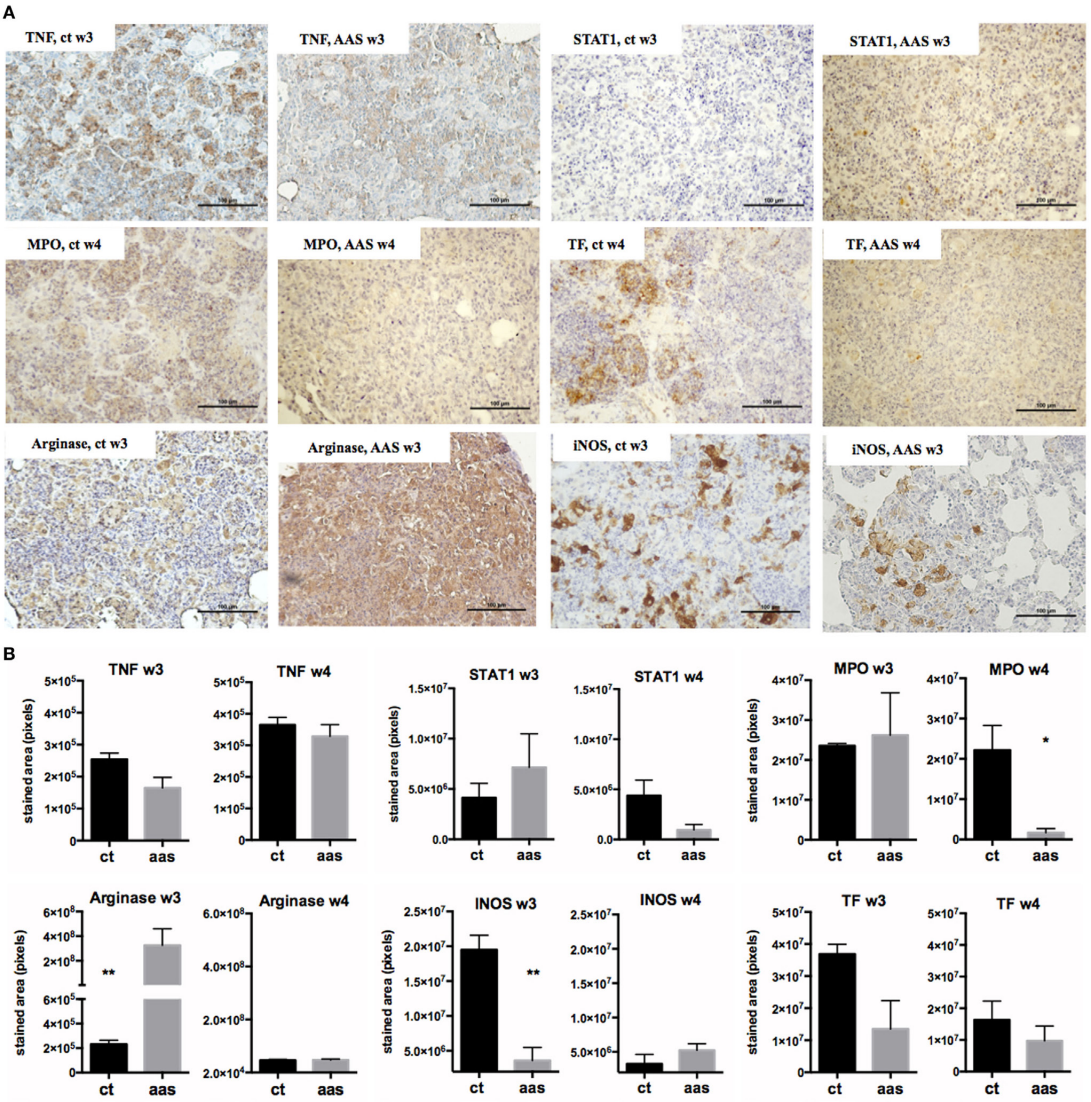
Impact of AAS in lung measured by IHQ. Panel **(A)** shows some pictures of the staining. The bar represents 100 µm. Panel **(B)** shows the results of the staining quantification by experimental group and timepoint. Abbreviations: AAS, aspirin; TNF, tumor necrosis α; STAT1, signal transducer and activator of transcription 1; MPO, myeloperoxidase; INOS, inducible nitric oxide synthase; TF, tissue factor.

**Figure 5:**
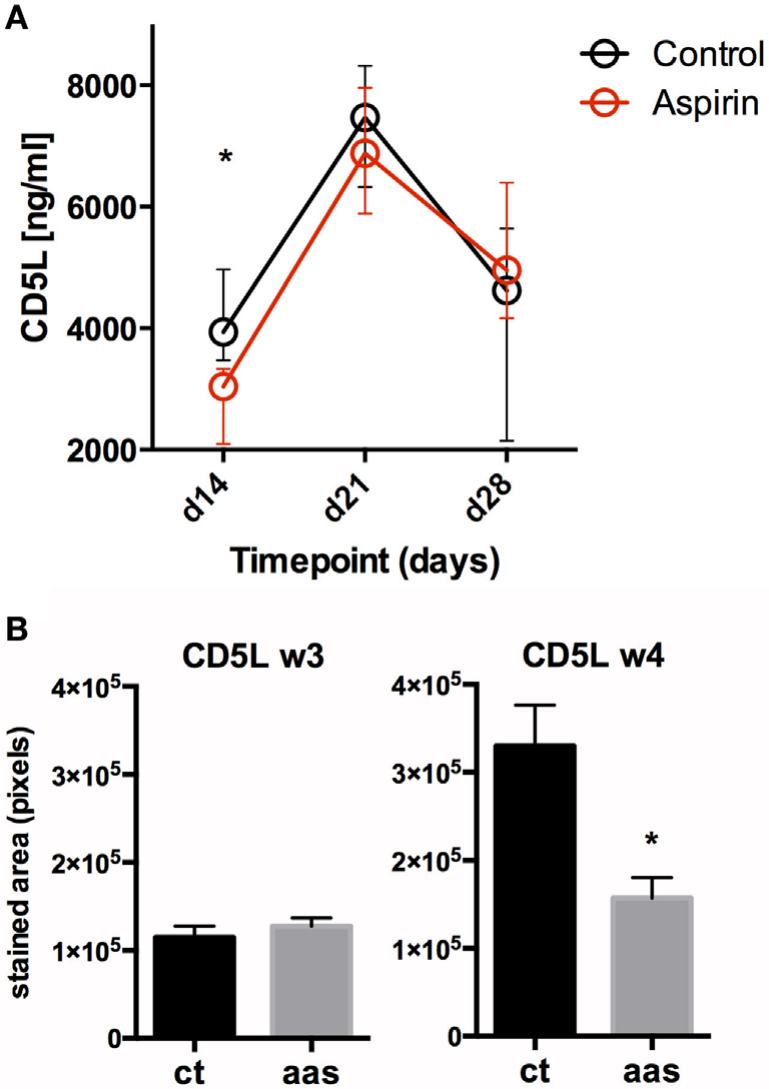
Impact of AAS on CD5L at a systemic and local level. Panel **(A)** shows serum levels (in pg/mL) for CD5L over time. Panel **(B)** shows CD5L in lung tissue on days 21 (week 3) and 28 (week 4) after challenge. Abbreviations: Ct, control; AAS, aspirin; d, day; w, week.

Figure 3 shows the results of the Luminex assay in serum for the cytokines. All median serum concentrations (pg/mL, plus 25–75% confidence intervals) for all cytokines/chemokines and CD5L measured by Luminex and ELISA at weeks 2 (day 14), 3 (day 21), and 4 (day 28) post-challenge are shown in Table S1 in Supplementary Material. The IHC values in lungs are shown in Figure 4, and the results of CD5L measurements in serum (measured by ELISA, Figure 5A) and lungs (assessed by IHC, Figure 5B) are shown in Figure 5.

#### Neutrophil Recruitment (G-CSF, KC, and MIP-2)

Our findings in serum (Figure 3) underline a general increase in the recruitment of granulocytes during TB, as the G-CSF and KC (CXCL-1) values for infected groups differed from the negative control in a statistically significant manner. As regards G-CSF serum levels, there were no statistically significant differences between treatment groups, although LDA reduced serum levels in late-stage disease. For KC, serum levels seemed to reach a plateau in week 3 in LDA-treated mice, while in the untreated infected animals (positive control) they appeared to keep rising through week 4, although the difference was not statistically significant. MIP-2 (CXCL2) levels in infected groups remained similar to those in healthy mice, except for week 4 (day 28), when the levels in the positive control animals increased (difference not statistically significant).

#### Acute-Phase Reaction: IL-1α, IL-1β, IL-6, and TNFα

Our results (Figure 3) suggest an expected systemic (acute-phase) inflammatory reaction in TB. Thus, IL-1α values in infected groups were below the range in healthy animals (not statistically significant), and levels in the LDA group were lower than those obtained from the positive control group on days 14 and 28 post-infection. IL-1β levels in both infected groups increased over time, showing the greatest differences at week 4 (day 28) when compared to healthy animals. This rise seemed to be less pronounced in the LDA group, although no changes were found to be statistically significant. IL-6 and TNFα values in infected groups differed with respect to the negative control at all timepoints in a statistically significant manner. Indeed, there was a statistically significant increase in serum IL-6 levels over the course of infection, with this being mitigated in the AAS group in later stages. TNFα levels in the LDA group peaked at week 3 (day 21) post-infection in a statistically significant manner, but returned to day 14 levels by week 4 (day 28).

#### T-Cell Response: IL-2, IFNγ, and IL-17

Animals from both infected groups showed increased IFNγ values at weeks 2 and 3 (days 14 and 21) when compared to negative controls, with these levels decreasing to those of healthy animals by day 28 (week 4). AAS significantly delayed this decrease. Thus, on day 28 post-infection, the IL-17 values for infected groups were statistically significantly higher than those for the negative control. No differences were encountered between the control and AAS groups. Although serum IL-2 levels were generally low, they were elevated in the infected groups with respect to levels in healthy mice, dropping back over the course of infection. This effect was less evident in the AAS group (not statistically significant).

#### The Anti-Inflammatory Cytokine: IL-10

IL-10 levels in infected groups were higher at week 2 (day 14) post-challenge when compared to the negative control, subsequently decreasing to the level found in healthy animals by day 28. The drop in the AAS group in week 3 (day 21) was less pronounced (*p* = 0.01).

#### IHC Results

The IHC results (Figure 4) showed differences in terms of stained area in pixels when using anti-TNF and anti-STAT1 antibodies. These differences were observed at a tissue level both over time and when comparing the two experimental groups. The presence of TNF was slightly lower in the AAS group, especially at week 3 post-infection (difference not statistically significant). In contrast, STAT1 was found to be elevated at week 3 and decreased at week 4 in the AAS group with respect to the control group. MPO+ cells were statistically significantly decreased in the AAS group in late-stage disease (week 4). Differences were encountered for both arginase and INOS detection at week 3. Thus, while arginase staining increased, INOS staining decreased in the AAS group, with the differences being statistically significant in both cases. Tissue factor was found to be decreased in the AAS group at both weeks 3 and 4 post-infection, although this decrease was more important at week 3 and was not statistically significant at either timepoint.

#### CD5L/AIM

Significant changes in serum CD5L/AIM levels were observed in both experimental groups over the course of the infection, dominated by a pronounced peak in week 3 post-infection (day 21), followed by a decrease. Serum CD5L/AIM levels were lower for the LDA group at weeks 2 and 3, although the difference was only statistically significant in week 2 (*p* = 0.0159) (Figure 5A). The IHC study revealed less CD5L/AIM in lung sections from the AAS-treated group at week 4 (*p* = 0.031).

## Discussion

The present study provides information on different LDA administration regimens (3 mg/kg) and their effect in a murine model of active TB.

The effect of AAS obtained *in vivo* has been classically considered to depend on the doses used. Thus, while low doses are commonly known to have an antithrombotic effect, only intermediate doses (500 mg to 3 g) were classically considered to be anti-inflammatory (24). However, the literature on preclinical and clinical studies in sepsis (25) shows that LDA triggers lipoxin synthesis, thus mediating anti-inflammatory and inflammation-resolving effects (26). Our results proved that maintained LDA also had an anti-inflammatory effect in the active TB model.

In our hands, when given preventatively or therapeutically in the absence of any other treatment, LDA statistically significantly increased survival in C3HeB/FeJ mice infected with TB, even enhancing the effect of RIMSTAR treatment. This is important as it stands in contrast with other findings, where AAS was described to antagonize the action of isoniazid (27) (which might raise the fear of the drug interfering with the standard treatment), and thus supporting the feasibility of AAS as an adjunct to TB drug therapy. Our findings, therefore, support the feasibility of AAS as an adjunct to TB drug therapy and provide evidence for the beneficial effects of LDA being mediated by an anti-inflammatory and an anti-mycobacterial mechanism, thereby reducing lung pathology over the course of the infection and BL in lungs, at least in late-stage TB.

As regards lung pathology, preventive treatment with LDA alone statistically significantly mitigated and delayed excess pulmonary damage in C3HeB/FeJ mice when compared to controls. Our results suggest that BCG vaccination can indeed prevent or delay terminal pathology due to *M. tuberculosis* infection in C3HeB/FeJ mice, and that treatment with LDA given therapeutically in addition to BCG vaccination does not provide any additional benefit, at least in the 4 months of this study. To determine whether LDA, administered therapeutically either alone or in combination with RIMSTAR or BCG vaccination, can statistically significantly mitigate and delay pulmonary damage during the course of infection, further experiments should be carried out using the same model, but with an experimental design considering several scheduled timepoints.

Analysis of serum levels for selected cytokines/chemokines showed that, as expected, TB itself generates a systemic inflammatory reaction with elevated levels of major neutrophil recruitment factors. Indeed, our results showed a significant increase in serum G-CSF, KC, IL-6, TNFα, and IL-17 levels when compared to negative controls. In contrast, the infection alone showed an inadequate drop in IFNγ, IL-2, and IL-10 levels over the course of infection. We observed a pronounced increase in serum IL-17 levels in late-stage disease, with this increase correlating with a decrease in IL-10 and CD5L. CD5L usually limits IL-17 production in Th17 and enhances IL-10 production in Th17 (28), and IL-10 has been suggested to play a role in controlling the anti-microbial activity and subsequent pulmonary tissue caseation (29). The observed late-stage rise in IL-17, therefore, probably reflects an increase in pathogenic Th17, thus leading to an increase in PMN infiltration and possibly explaining the excess late-stage lung pathology.

Overall, and although not reaching statistical significance for all measurements, our results suggest a systemic anti-inflammatory effect due to AAS treatment, as also described by Marzo et al. and Vilaplana et al. for ibuprofen in the same mouse model (12, 23). First, our findings point toward a systemic modulating effect of LDA on systemic neutrophil recruitment, thus resulting in a reduction in serum G-CSF and KC levels in late-stage disease. The IHC results confirmed the presence of fewer neutrophils in lungs, with a statistically significant decrease in MPO+ staining being observed at day 28 (week 4). With regards to the effects on neutrophil recruitment, it has been suggested that the anti-inflammatory mechanism of action of LDA is mitigated by both the reduced number (recruitment) of neutrophils and by reduced prostaglandin production in monocytes, lymphocytes, and neutrophils, which is implicated in the origin and development of granulomas (8, 14). These cells possess COX-2 activity and produce the prostaglandins (PG) implicated in the inflammation and lung damage associated with active TB, although in the short-term PGE2 might be beneficial (8). Moreover, the results for the AAS group showed less acute phase reaction cytokines (IL-6, IL-1β, and TNF-α) in the later stages. It has been observed that increased IL-6 levels directly correlate with increased BL (30) and X-ray severity in active TB in humans, therefore, this could be a valuable marker for predicting response to anti-TB treatment, with a pronounced reduction of serum levels being observed following treatment (31). Serum IL-1β levels in infected mice rose above the levels for healthy mice in late-stage disease. It has been described that increased IL-1β levels directly correlate with X-ray severity in active pulmonary TB in humans (31), and increased lung pathology was also observed in our study over the course of infection. Consequently, the lower levels achieved with LDA could be a reflection of the reduced lung pathology found in late-stage TB in the animals in this group.

Eisen et al. previously suggested that the beneficial anti-inflammatory effect of AAS might be mediated by a balancing effect on TNFα and that this could only be achieved at high dose, commonly known as the anti-inflammatory dose (32). Our results suggest that LDA does indeed balance TNF-α in TB in C3HeB/FeJ mice, significantly increasing serum levels in earlier stages (week 3) and reducing serum levels in the later stages of infection compared to non-treated positive controls. However, this increase could also be a consequence of the increase in BL at week 3 observed in the AAS group. Indeed, according to our IHQ results, TNF-α was slightly decreased in lung tissue (especially at week 3). However, the TNF-α increase observed at a serum level was not associated with worsened lung damage at that precise timepoint or subsequently according to the pathology results (33). The increase detected in serum levels at week 3 in the AAS group might enhance the Th1 response, the macrophage response and, consequently, clearance of the mycobacteria, acting synergistically with IL-2 and IFNγ. Increased levels of IFNγ and IL-2 have been found to be associated with moderate cases of human TB rather than more severe cases (34). Indeed, with LDA treatment we see a delay in the decrease in T cell response at a serum level (IFN-γ, IL-2, and IL-10) observed over the course of the infection, which reaches statistical significance at week 3. This delay at this precise timepoint might be responsible for controlling the tissue damage and BL by week 4, when infected animals are usually critically ill because of TB.

Signal transducer and activator of transcription 1 (STAT-1) was found to be elevated in lung tissue from LDA-treated animals compared to controls at week 3 and decreased at week 4, thus mirroring the results obtained in serum. The decrease in serum IL-10 levels over the course of the infection was also delayed in LDA-treated mice, and although the AAS group showed the lowest values in late-stage disease, the anti-inflammatory effect observed, as represented by higher IL-10 levels at week 3, could be considered a problem. It has been found that high serum IL-10 levels in patients with pulmonary TB lead to a slower response to treatment and a lack of bacterial control, thereby underlining the importance of a strong pro-inflammatory response when lung tissues are still intact (30, 35). In addition, reduced IL-10 (and IL-4) levels are associated with more moderate cases of human TB (34). In our study, the impact of the anti-inflammatory effect achieved by LDA seems to be beneficial rather than detrimental as, overall, AAS manages to decrease the inflammation in late-stage disease without impairing the T cell response in the short term. At a tissue level, we were able to demonstrate an increase in arginase-1 and a decrease in INOS at a lung level by week 3 in animals treated with LDA compared to controls. Arginase and INOS have been reported to be markers for characterizing and differentiating different macrophage functions as pro-healing/anti-inflammatory (Arginase) or pro-inflammatory (INOS) in the context of TB (36). As such, LDA treatment may be temporarily switching the phenotype of lung macrophages toward a more pro-healing/anti-inflammatory mode at week 3, which could have an important impact on disease outcome.

As regards CD5L measurements, mice from the AAS group had lower values than those from the control group at early timepoints (weeks 2 and 3) in serum and at later stages (week 4) at a lung level. CD5L/AIM is an innate protein that has been previously described to peak in plasma at week 3 post-infection, which may reflect a strong early innate immune response aimed at controlling *M. tuberculosis* growth. This enhances autophagy for elimination of the bacilli and acts in an anti-inflammatory (regulatory) manner on macrophages and Th1 (28, 37, 38). According to our results, the observations at a serum level may not be mirrored at the lung level as we see increased CD5L in lungs by IHC in week 4 (less pronounced in AAS-treated animals). This could suggest that this protein may play a role both during early infection and also in late stages of the disease, a point that should be studied in further experiments.

Finally, the measurement of TF by IHQ showed high levels at week 3, which was diminished by AAS treatment. TF is the principal initiator of the coagulation cascade, which plays a role in inflammation (39) and innate immunity and is expressed in response to different stimuli, such as infections and inflammation (40). The role of TF in TB is controversial. Thus, while some authors have stated that although this factor is increased, TF-mediated coagulation does not contribute to the host’s defense (41), others have suggested a protective role for TF during *M. tuberculosis* infection by demonstrating, in a mouse model of TB, that TF deficiency is associated with increased *M. tuberculosis* replication in lungs (42). LXA4 was found to be able to promote a proinflammatory and prothrombotic profile *in vitro* by inducing TF expression (43). However, a tendency toward hypercoagulation is a known problem in TB patients, especially in severe cases, therefore, the modulation of this state has been proposed as a target for AAS treatment in TB meningitis (9, 44). Indeed, our results point in this direction.

Our results are in contrast to some previous *in vitro* and *in vivo* studies in the literature, which suggest that inhibition of prostaglandin pathways involving a decrease in PGE2 and production of LXA4 is detrimental in terms of immunity to TB and might impair T cell immunity (45–47). In our opinion, the coexistence of contradictory results in the literature in this sense is probably due to the important matter of timing in HDT administration during TB, as a strong inflammatory response is needed at the beginning of the infection for possible control or even clearance of the mycobacteria. Our study was performed using a murine model based on intravenous infection of C3HeB/FeJ mice, which consistently develop lesions with liquefactive necrosis after infection with *M. tuberculosis* and die after 30 days in the absence of treatment. The characteristics of these lesions well mimic active TB in humans at diagnosis (when the disease is exacerbated, with an important inflammatory component and subsequent host tissue destruction), but not human latent infection or disease without a predominant inflammatory response. For this reason, we consider that results from studies in C3HeB/FeJ mice are not applicable to immune-competent humans chronically infected with *M. tuberculosis* or suffering from mild forms of TB. In light of the above, and as a conclusion to our work, we propose that LDA should only be considered as an adjunct to antibiotic treatment and/or vaccination in patients who present with clinical signs of disease (wasting, cough, etc.). On the other hand, the finding that preventative treatment with LDA increases survival, delays excess granuloma formation in lungs, and reduces BL in late-stage TB disease in our model suggests LDA could be given to patients at high risk for TB as a preventive measure, although further studies should be performed to confirm this point.

## Ethics Statement

All procedures were performed according to protocol DMAH6119, which was reviewed by the Animal Experimentation Ethics Committee of the Hospital Universitari Germans Trias i Pujol (registered as B9900005) and approved by the Dept d’Agricultura, Ramaderia, Pesca, Alimentació i Medi Natural of the Catalan Government, according to current national and European Union legislation regarding the protection of Experimental animals. Mice were supervised daily following a strict monitoring protocol in order to ensure animal welfare, and euthanized, if required, with isoflurane (inhalation excess).

## Author Contributions

CV headed the project. CV, MS, and P-JC conceived and planned the experiments. VK, PR-M, MM-C, GT, EG, and JD carried out the experiments. VK, GT, MS, P-JC, and CV contributed to the interpretation of the results. VK and CV took the lead in writing the manuscript. All authors provided critical feedback and helped to shape the research, analysis, and manuscript.

## Conflict of Interest Statement

The authors declare that the research was conducted in the absence of any commercial or financial relationships that could be construed as a potential conflict of interest.
