# Supplementary material for: A Beneficial Effect of Low-Dose Aspirin in a Murine Model of Active Tuberculosis
Source: Front Immunol. 2018 Apr 23;9:798. doi: 10.3389/fimmu.2018.00798 (PMC5924809; doi:10.3389/fimmu.2018.00798)
Supplement: Supplementary file 1 [file table_1.docx]

|  |  |  |  |  |  |  |  |  |  |
| --- | --- | --- | --- | --- | --- | --- | --- | --- | --- |
|  |  | **d14** | | **d21** | | **d28** | | **Negative control** |  |
|  |  | **ct** | **AAS** | **ct** | **AAS** | **ct** | **AAS** |  |  |
|  | **G-CSF** | 1333 (849.7-1554) | 818 (701.5-1156) | 4093 (2948-6167) | 7079 (3596-9303) | 71119 (33595-90476) | 48216 (7093-62834) | 410.5 (337-565.5) |  |
|  | **KC** | 587.9 (379.2-868.9) | 468.7 (267.2-607.5) | 1484 (1229-2268) | 2446 (1977-2992) | 3006 (1425-4532) | 1983 (1029-3427) | 145.9 (96.8-280.3) |  |
|  | **MIP-2** | 345.3 (275.4-432.3) | 333.6 (237.4-499.9) | 289.4 (195.9-376.8) | 352.6 (313.6-412.5) | 391.6 (325.3-439.6) | 329.3 (281-398.3) | 364 (273.3-393.3) |  |
|  | **IL-1-α** | 424.5 (182.3-616.5) | 227.3 (117.6-372.7) | 474.6 (362.7-582.7) | 377.6 (306.5-684.1) | 426 (317.7-434.1) | 304.7 (144.3-372.9) | 715.8 (338.8-1019) |  |
|  | **IL-1-β** | 8.07 (3.2-43.5) | 3.2 (3.2-30) | 5.635 (3.2-36.1) | 23.3 (4.4-39.6) | 63.3 (49.1-68.2) | 24.65 (3.2-66.1) | 8.6 (3.2-47.5) |  |
|  | **IL-6** | 128.2 (95.25-147.8) | 84.71 (77.3-96.9) | 533.7 (436-759.9) | 472 (326.6-583.3) | 1986 (1358-4551) | 1214 (726-1930) | 19 (13-59.2) |  |
|  | **TNF-α** | 48.39 (47.15-52.71) | 47.8 (36.4-50.5) | 61.44 (57.9-75.9) | 105.3 (80-122) | 74.89 (56.5-88.01) | 53.34 (40.8-71.1) | 29.2 (17.3-32.7) |  |
|  | **IL-2** | 24.9 (3.2-26.85) | 18.86 (7.4-38.7) | 12.66 (3.2-20) | 18.26 (4.6-39.9) | 3.2 (3.2-3.2) | 4.645 (3.2-29.3) | 3.2 (3.2-3.2) |  |
|  | **IFN-γ** | 369.3 (299.7-472.1) | 376.1 (259.6-608.5) | 226.6 (159.7-292.9) | 423.4 (380.7-601.7) | 3.2 (3.2- 76.6) | 53.37 (28.7-88.7) | 3.2 (3.2-21.7) |  |
|  | **IL-17** | 3.2 (3.2-3.415) | 3.2 (3-3.2) | 6.815 (4.2-10.3) | 3.82 (3.2-9.61) | 22.22 (17.8-39.5) | 22.86 (10-41.6) | 3.2 (3.2-6.8) |  |
|  | **IL-10** | 25.67 (20.96-28.6) | 26.2 (20.9-28.5) | 7.265 (5.8-10.5) | 15.56 (12.6-21.3) | 15.55 (3.2-19.2) | 8.645 (4.9-13.2) | 11.25 (6.7-25.1) |  |
|  | **CD5L** | 3938 (3473-4964) | 3040 (2096-3334) | 7470 (6328-8319) | 6878 (5886-7961) | 4619 (2149-5641) | 4955 (4168-6395) |  |  |
|  |  |  |  |  |  |  |  |  |  |

**Supplementary Table 1. *Results of the immunological assays (Luminex/ELISA).***

Median serum values (with 25%-75% Interval of Confidence) for cytokines/chemokines and CD5L are shown in pg/mL. Ct: Control group. AAS: Aspirin. Negative Control: healthy animals. D: day post-infection.
